# Silver-Based Filler Silicone Rubber Composites for Electromagnetic Interference Shielding Applications

**DOI:** 10.3390/polym18141713

**Published:** 2026-07-12

**Authors:** Yilin Liu, Zhe Chen, Jinlei Qu, Baogang Zhang, Le Kang, Yongtao Qu

**Affiliations:** 1 Engineering Research Center for High Performance Polymer and Molding Technology, Ministry of Education, Qingdao University of Science & Technology, Qingdao 266042, China; 15864715108@163.com (Y.L.); anxiangshuying1223@163.com (Z.C.); sdqujinlei@hotmail.com (J.Q.); zbg0302@163.com (B.Z.); 2 School of Engineering, Physics and Mathematics, Ellison Building, Northumbria University, Newcastle upon Tyne NE1 8ST, UK; 3 School of Photovoltaic and Renewable Energy Engineering, University of New South Wales, Sydney, NSW 2052, Australia

**Keywords:** silicone rubber, silver-plated fillers, electromagnetic interference, percolation threshold, sandwich structure

## Abstract

Electromagnetic interference (EMI) shielding materials are critical for reducing EMI pollution and enhancing information security. This study presents a systematic comparison of silver-plated copper (Cu@Ag; flake-like morphology; the average particle size D50 = 20.1 μm) and silver-plated aluminium (Al@Ag; spherical morphology; D50 = 47.5 μm) fillers with distinct morphologies incorporated into silicone rubber matrices via Rheomixer blending, open-mill compounding, and peroxide vulcanisation. This work aims to elucidate how filler morphology and multilayer sandwich architecture govern shielding efficiency and related material properties. The flake-like Cu@Ag fillers demonstrated superior low-loading performance. Due to their high aspect ratio and enhanced interfacial contact, Cu@Ag composites reached a critical loading for practical EMI shielding performance at 150 phr. In contrast, spherical Al@Ag fillers required a higher loading of 200 phr to achieve the same effect. Both composites achieved EMI shielding effectiveness exceeding 90 dB at 250 phr filler loading across the X-band frequency range (8.2–12.4 GHz). Innovatively, sandwich-structured composites were fabricated by combining Cu@Ag and Al@Ag layers through co-vulcanization, achieving approximately 110 dB shielding effectiveness, which is a ~33% improvement over single-layer composites at equivalent filler loading (200 phr). Analysis of the shielding mechanisms reveals that this enhancement results from multiple electromagnetic wave interactions, including increased reflection losses at morphologically distinct layer interfaces and enhanced absorption through conductivity gradients. This work demonstrates that a rational combination of flake-like and spherical fillers with contrasting morphologies and conductivity characteristics in multilayer architectures provides a powerful strategy for developing high-performance flexible EMI shielding materials.

## 1. Introduction

With the rapid advancement of modern electronic information technologies, electromagnetic interference (EMI) has emerged as a critical threat to electronic information security, the safety of precision instruments and human health. Research indicates that electromagnetic waves may adversely affect human nerve cells, brain tissue, immune system, and metabolism [1], potentially increasing cancer risk [2]. Moreover, EMI pollution disrupts the normal operation of sensitive electronic devices. These concerns have consequently driven significant research interest in developing high-performance EMI shielding materials [3,4,5]. In recent years, conductive polymer composites have attracted considerable attention due to their tuneable EMI shielding properties, corrosion resistance, ease of processing, and lightweight characteristics [6,7]. The application environments of EMI shielding materials are complex, requiring consideration of not only EMI shielding performance but also extreme temperatures and aging resistance. Silicone rubber, characterised by its siloxane (Si-O) backbone, exhibits excellent resistance across a broad operational temperature range (typically from −55 °C to 200 °C) for continuous service, with thermal stability exceeding 300 °C [8,9], photo-oxidative ageing resistance, and biocompatibility. While prolonged thermo-oxidative exposure can induce chain scission under oxygen-rich environments at elevated temperatures [10], post-curing treatment promotes additional cross-linking of residual reactive sites that enhances long-term oxidative stability [8], making silicone rubber an attractive and well-established matrix for advanced EMI shielding applications. However, the inherent electrical insulating characteristics of silicone rubber severely limit its EMI shielding effectiveness (SE). This necessitates the incorporation of functional fillers to establish conductive networks, thereby achieving enhanced shielding performance. The type, morphology, and dispersion state of conductive fillers in the polymer matrix are critical factors determining the electromagnetic shielding performance of silicone rubber composites.

Carbon-based fillers (e.g., carbon nanotubes, graphene, and carbon fibres) have made significant advancements for EMI shielding applications. Zhang et al. developed carbon black/silicone rubber composites with a shielding effectiveness exceeding 20 dB across a frequency range of 0.01 MHz to 10 GHz at low filler loadings [11]. Park et al. incorporated two-dimensional graphene sheets into silicone rubber to fabricate a multifunctional graphene/silicone rubber composite film via hot pressing, which exhibited an electromagnetic shielding effectiveness of 25 dB across the 1.0 to 3.0 GHz frequency range [12]. The effectiveness of carbon-based fillers in establishing conductive networks is strongly governed by their percolation threshold, which is the minimum concentration that a continuous conductive pathway forms throughout the polymer matrix. Owing to their high aspect ratio and large specific surface area, nanoscale carbon-based fillers such as MWCNTs and graphene achieve relatively low percolation thresholds, typically in the range of 0.2–3 vol% for CNTs [13] and 0.3–0.5 vol% for graphene [14] in polymer matrices. Carbon black, by contrast, requires higher loadings of approximately 3–10 wt% to reach percolation due to its lower aspect ratio and tendency to agglomerate [15]. Nevertheless, carbon-based fillers exhibit limited effectiveness in high-frequency bands.

In comparison, metallic fillers including iron (Fe), aluminium (Al), nickel (Ni), copper (Cu), silver (Ag) and others demonstrate superior broadband performance in EMI SE. However, they face challenges such as oxidation susceptibility and high costs. Advancements in coating technology have enabled the development of composite conductive fillers, such as silver-coated glass microspheres, silver-plated aluminium powder (Al@Ag), silver-plated copper powder (Cu@Ag), and nickel-coated carbon materials [16,17,18]. These composite conductive fillers offer high conductivity, oxidation resistance, relatively low density and cost-effectiveness. However, due to the micron-scale particle size and spherical or near-spherical morphology of most metal-plated fillers, significantly higher filler loadings are typically required to reach percolation compared to nanoscale carbon fillers. For instance, spherical metal particle-filled composites commonly require substantially higher filler loadings to establish a conductive network, while high-aspect-ratio metallic flake fillers can reduce the percolation threshold considerably through enhanced inter-particle contact and overlapping pathways [18]. This morphology-dependent percolation behaviour directly determines the techno-economic feasibility of the composite: lower percolation thresholds translate to reduced filler content, lower material costs, and better-preserved mechanical properties of the rubber matrix. Furthermore, combining carbon nanomaterials with metal microparticles in hybrid filler systems has been shown to achieve comparable or superior electrical performance at significantly reduced total filler loadings. For instance, 3 wt% MWCNTs combined with only 5 wt% Cu or Ni microparticles in silicone rubber yielded optimised electrothermal performance [19]. Xu et al. developed lightweight silver-coated glass microspheres (Ag@GMs)/silicone rubber composites, achieving a remarkable electromagnetic shielding effectiveness of 120 dB at X-band frequencies (8.2–12.4 GHz) [20]. Wang et al. fabricated nickel-coated carbon fibre (Ni@CF)/silicone rubber composites with an electromagnetic shielding effectiveness of approximately 80 dB across a low-frequency range, from 30 MHz to 1.2 GHz [21], demonstrating the versatility of metal-coated filler systems across different frequency regimes. While these single-layer metal-filled composites exhibit excellent shielding performance, achieving such high effectiveness typically requires substantial filler loadings and relatively thick composite layers, which may limit their application in weight-sensitive and space-constrained scenarios.

Beyond the inherent properties of the filler, EMI SE can be further enhanced through structural design [22,23,24]. Designing multilayered functional structures increases the reflection loss of electromagnetic waves within the material, thereby improving its EMI SE. Wei et al. developed sandwich-structured Fe_3_O_4_@CNT/NR composites exhibiting low thermal conductivity and high EMI SE [25]. Wang et al. fabricated a novel flexible sandwich-structured silicone rubber/graphene composite, achieving a maximum EMI SE of 30.42 dB, representing a 59.6% improvement over the homogeneous-structured composites [26].

Despite these advances, there remains a need to systematically compare different metal-plated fillers and optimize structural designs for enhanced EMI shielding performance in silicone rubber matrices. Furthermore, the mechanisms underlying the superior performance of sandwich structures require deeper investigation. This study therefore aims to address these gaps through the following specific tasks: (1) a systematic characterisation of the morphology and particle size distribution of Cu@Ag and Al@Ag fillers and their influence on composite microstructure; (2) a comparative investigation of the effects of filler type and loading on the physical, mechanical, thermal, electrical, and EMI shielding properties of single-layer composites; (3) fabrication and a characterisation of sandwich-structured multilayer composites combining Cu@Ag and Al@Ag layers via pre-forming and co-vulcanisation; and (4) elucidation of the mechanisms responsible for the enhanced EMI shielding performance of sandwich structures relative to single-layer composites. This work aims to provide a reference for the preparation of high-performance flexible silicone rubber EMI shielding materials and elucidate the mechanisms responsible for enhanced performance in multilayer structures.

## 2. Materials and Methods

### 2.1. Materials and Apparatus

Vinyl methyl silicone rubber (50° Shore A hardness, pre-mixed, Brand 9150, Shandong Dongyue Silicone Co., Ltd., Zibo, Shandong, China) was selected as the primary matrix for the following reasons. First, the pre-mixed Grade 9150 compound incorporates a reinforcing fumed silica filler with optimised surface treatment, providing a well-characterised and highly reproducible baseline matrix that minimises batch-to-batch variability. This is an important consideration given the broad filler loading range (0–250 phr) investigated in this study. Second, its 50° Shore A hardness and associated rheological characteristics, particularly its reduced viscosity at the Haake rheomixer processing temperature of 70 °C, ensure adequate processability and homogeneous filler dispersion even at the highest filler loadings studied (250 phr). Third, Grade 9150 is widely used in industrial silicone rubber product manufacturing in China, meaning the results of this study have direct relevance to practical manufacturing contexts. The methyl vinyl silicone rubber (Brand 110-3, Zhejiang Hesheng Silicon Co., Ltd., Jiaxing, Zhejiang, China) was blended with Grade 9150 at a 40:60 ratio to further optimise processability and mechanical properties across the full filler loading range. 2,5-Dimethyl-2,5-bis(tert-butylperoxy)hexane (Bis-2,5), industrial grade with 50% active content, was used as the curing agent. Silver-plated copper powder (Cu@Ag, Product No. Li-0120x) and silver-plated aluminium powder (Al@Ag, Product No. AL-20S23) were supplied by Shenzhen Lihongjin Technology Co., Ltd., Shenzhen, Guangdong, China. The morphology and the size distribution of both fillers are studied in Section 3.1.

A double-drum open mill, Model XSK1608, was obtained from Shanghai Rubber Machinery Factory., Shanghai, China. A rubber processing analyser, Model RPA2000, was sourced from Alpha Technologies, Akron, Ohio (OH), USA. A plate vulcanizing machine, Model XLB-D 400 × 400, was provided by Zhejiang Huzhou Dongfang Machinery Co., Ltd., Huzhou, Zhejiang, China. An electric blower dryer, Model DGG-9070B, was supplied by Shanghai Senxin Experimental Instrument Co., Ltd., Shanghai, China. A pneumatic punching machine, Model MZ-4102B, and a rubber hardness tester, Model LX-A, were acquired from Jiangdu Pearl Machinery Co., Ltd., Yangzhou, Jiangsu, China. A field emission scanning electron microscope (SEM), Model JSM-7500F, was obtained from JEOL Ltd., Tokyo, Japan. A tensile testing machine, Model AI-7000S, was sourced from Gotech Testing Machines Inc., Taichung, Taiwan, China. An ion sputtering instrument, Model SBC-12, was provided by Beijing Zhongke Keyi Co., Ltd., Beijing, China. A broadband dielectric impedance spectrometer, Model BDS 40, was supplied by Novocontrol Technologies GmbH, Montabaur, Germany. A vector network analyser, Model ZNB-20, was acquired from Rohde & Schwarz GmbH, Munich, Germany.

### 2.2. Sample Preparation

Cu@Ag/silicone and Al@Ag/silicone EMI shielding composites were fabricated by conventional rubber processing techniques. Single-layer composites were prepared across a filler loading range of 0–250 phr, and sandwich-structured multilayer composites were fabricated via pre-forming and co-vulcanisation, as detailed in Section 2.2.1, Section 2.2.2 and Section 2.2.3 below.

#### 2.2.1. Composite Formulation

The composites consisted of 60 g of 50 Shore A pre-mixed vinyl methyl silicone rubber, 40 g of methyl vinyl silicone rubber, and 1 g of 2,5-dimethyl-2,5-bis(tert-butylperoxy)hexane (Bis-2,5) as the curing agent. The Cu@Ag or Al@Ag filler loading was systematically varied at 50, 100, 150, 200, and 250 parts per hundred rubber (phr). Unfilled silicone rubber composites (designated as Cu@Ag-0 and Al@Ag-0 with 0 phr filler loading) were prepared as control samples to establish baseline properties and evaluate the effect of metal fillers on thermal stability, electrical conductivity, and EMI shielding performance. The broad filler loading range of 0–250 phr was selected to systematically characterise the full percolation behaviour of both Cu@Ag and Al@Ag filler systems and to achieve the target EMI shielding effectiveness exceeding 90 dB required for high-demand aerospace and electronic shielding applications. While high filler loadings are acknowledged to increase agglomeration tendency and reduce mechanical properties, these effects were mitigated through optimised open-mill processing at low shear rates and the use of morphologically complementary filler combinations in sandwich structures, which help achieve superior shielding performance at acceptable mechanical properties.

#### 2.2.2. Mixing Process

**Figure 1:**
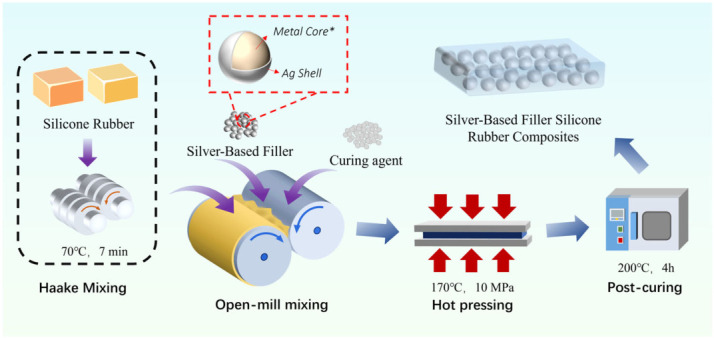
The preparation of metal filler/silicone rubber composites. * The metal core material is either Cu or Al. It should be noted that this schematic is intended solely to demonstrate the core-shell configuration. The morphologies of both fillers are exemplified in Figure 2.

Figure 1 illustrates the sample preparation procedure. Initially, both types of silicone rubber were blended in a Haake rheomixer at 70 °C for 7 min. The mixture was then processed on an open mill where the curing agent was incorporated. Subsequently, fillers were added at predetermined ratios. The mixing process was kept consistent across all composite materials to ensure reproducibility.

**Figure 2:**
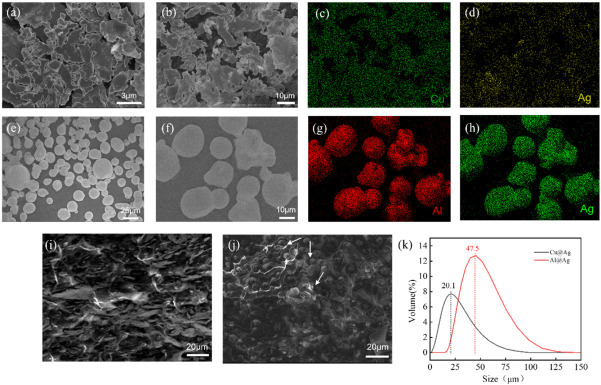
Morphological characterisation and elemental analysis of metal fillers. SEM images of Cu@Ag at (**a**) 1000× and (**b**) 3000× magnification showing its flake-like morphology; corresponding elemental mapping of (**c**) Cu and (**d**) Ag distribution. SEM images of Al@Ag at (**e**) 400× and (**f**) 2500× magnification showing spherical/ellipsoidal morphology; corresponding elemental mapping of (**g**) Al and (**h**) Ag distribution. Cross-sectional SEM images of composites with 150 phr filler loadings of (**i**) Cu@Ag-150 and (**j**) Al@Ag-150 respectively. The white arrows in figure (**j**) indicate the spherical/ellipsoidal Al@Ag fillers in the composite. (**k**) Particle size distribution of Cu@Ag and Al@Ag.

The preparation process detailed in Figure 1 was determined through a series of systematic preliminary experiments designed to optimize the balance between filler dispersion, final composite performance, and overall processability across the broad filler loading range (0–250 phr) studied.

Specifically, the processing of silicone rubber products typically involves three stages: compounding, vulcanization, and post-curing.

(1) Compounding:

Base compounding was carried out in a Haake internal mixer at 70 °C for 7 min. A temperature of 70 °C appropriately reduces the viscosity of the 50 Shore A pre-mixed vinyl methyl silicone rubber compound, facilitating uniform blending with methyl vinyl silicone rubber. Preliminary experiments showed that mixing times shorter than 5 min resulted in incomplete homogenisation of the two rubber grades, as evidenced by non-uniform torque profiles. However, mixing times exceeding 10 min caused the compound temperature to rise beyond 80 °C, risking premature activation of the peroxide curing agent (Bis-2,5; initial decomposition temperature, ~80 °C) and partial cross-linking during compounding. The 7 min duration therefore represents the optimal balance between thorough homogenisation and prevention of premature vulcanisation. To prevent localized overheating during internal mixing, the fillers and curing system were incorporated on an open mill rather than in the internal mixer. Localized overheating can arise from high shear and frictional forces in the internal mixer. If the compound temperature exceeds the initial decomposition temperature of the curing agent, partial cross-linking may occur during compounding, leading to premature vulcanisation. Furthermore, the high shear forces in the internal mixer may damage the silver coating of the metal fillers, compromising their electrical conductivity and EMI shielding performance. Open-mill processing operates at significantly lower shear rates, making it more suitable for incorporating both the curing system and the sensitive silver-coated fillers. The compound was removed from the internal mixer and transferred to a two-roll open mill for further mixing. After the compound wrapped around the front roll, the curing system, 2,5-Dimethyl-2,5-bis(tert-butylperoxy)hexane, was added. The silicone rubber was repeatedly folded and milled until the curing agent was uniformly dispersed, resulting in a well-mixed masterbatch. Subsequently, different proportions of silver-coated fillers were added and mixed under consistent processing conditions and time to ensure uniformity.

(2) Vulcanization:

Vulcanization characteristics were measured using a rubber process analyser (RPA) according to ASTM D5289 [27] at 170 °C under 10 MPa pressure. The composites were compression moulded into 2 mm sheets at 170 °C under 10 MPa pressure, with the curing duration set to t_c_90 + 2 min as determined by moving die rheometer analysis. A temperature of 170 °C is typical for peroxide-based vulcanization systems. At this temperature, the vulcanization rate is sufficiently fast to ensure production efficiency while maintaining controllable cross-linking kinetics. The vulcanization time was set to t_c_90 + 2 min, which serves as a “golden rule” to ensure complete vulcanization. Here, t_c_90 represents the time required to achieve 90% cross-linking, and the additional 2 min (safety margin) ensures thorough curing even at the centre of thicker products.

(3) Post-Curing:

Post-curing was subsequently conducted in a forced-air circulation oven at 200 °C for 4 h to ensure complete cross-linking. A temperature of 200 °C is widely adopted in the silicone rubber literature for post-curing. Post-curing at 200 °C promotes further cross-linking of residual reactive sites, leading to a more stable cross-linked network. It is also the optimal temperature for facilitating the reaction between the structure control agent and the surface of fumed silica. Additionally, this step removes low-molecular volatiles generated during compounding and vulcanization (e.g., water and peroxide decomposition byproducts), thereby further enhancing the physical and mechanical properties of the silicone rubber. The post-curing duration of 4 h was determined through systematic trials (ranging from 2 to 8 h) as the time point that delivers optimal performance.

#### 2.2.3. Sandwich-Structure Preparation

Sandwich-structured composites were prepared using Cu@Ag/silicone and Al@Ag/silicone layers, each containing 200 phr of the respective fillers. The multilayer structures were fabricated through pre-forming and co-vulcanization processes. Test specimens with an approximately 2 mm total thickness were prepared featuring Al@Ag-Cu@Ag-Al@Ag- and Cu@Ag-Al@Ag-Cu@Ag-layer sequences, designated as M-3 and M-4, respectively. Single-layer composites with 200 phr Cu@Ag and Al@Ag were prepared as controls and labelled as M-1 and M-2, respectively.

### 2.3. Characterisation

#### 2.3.1. Physical and Mechanical Properties

Shore A hardness was measured using a rubber hardness tester (Model LX-A, Jiangdu Pearl Machinery Co., Ltd., Yangzhou, Jiangsu, China) in accordance with GB/T 531.1-2008 [28] on specimens of a 6 mm thickness. Five measurements were taken at different positions on each specimen and the average value reported.

Tensile properties were evaluated using a tensile testing machine (Model AI-7000S, Gotech Testing Machines Inc., Taichung, Taiwan, China) in accordance with ISO 37 [29] at a crosshead speed of 500 mm·min^−1^ using dumbbell-shaped Type 2 specimens. Five specimens were tested for each composition and the average values reported.

Thermal stability was assessed using a thermogravimetric (TGA) analyser at a heating rate of 10 °C·min^−1^ from 50 to 800 °C under a nitrogen (N_2_) atmosphere with a flow rate of 50 mL·min^−1^. The sample mass was approximately 10 mg for each measurement.

Dynamic mechanical properties were evaluated using a rubber processing analyser (Model RPA2000, Alpha Technologies, Akron, OH, USA) in accordance with ASTM D6204-2015 [30], with strain scanning conducted in shear mode at 60 °C, a strain range of 0.01% to 100%, and a frequency of 1 Hz.

#### 2.3.2. AC Conductivity

Circular specimens (Ø 25 mm; thickness, ~2 mm) were gold-coated on both sides using an SBC-12 ion sputtering coater to ensure good electrical contact. Electrical properties were characterised via broadband dielectric/impedance spectroscopy (BDS 40, Novocontrol Technologies GmbH, Montabaur, Germany) under the following conditions: 1 V AC amplitude over a frequency range of 10^−1^ to 10^7^ Hz at room temperature.

#### 2.3.3. Morphological Analysis

Cross-sectional morphology was examined by field emission scanning electron microscopy (SEM, Model JSM-7500F, JEOL Ltd., Tokyo, Japan) at an accelerating voltage of 20 kV. Specimens were prepared by cryogenic fracture in liquid nitrogen to preserve the filler distribution without mechanical deformation. The fractured cross-sections were mounted on aluminium stubs using conductive adhesive tape and sputter-coated with gold for 60 s using an ion sputtering instrument (Model SBC-12, Beijing Zhongke Keyi Co., Ltd., Beijing, China) to minimise charging artefacts. Elemental mapping was performed using the energy dispersive X-ray spectroscopy (EDX) detector integrated with the SEM system.

#### 2.3.4. Filler Size Distribution

The Mastersizer 3000+ Ultra laser diffraction particle size analyser (Malvern Panalytical, Malvern, UK) was used to understand the filler size distribution. Approximately 15 mg of the filler sample was dispersed in 50 mL of absolute ethanol and subjected to ultrasonic treatment (AK-040SD, 360W, Shenzhen Yujie Cleaning Equipment Co., Ltd., Shenzhen, Guangdong, China) for 10 min to break down any soft agglomerates. The suspension was then continuously stirred at 2000 rpm during the measurement to maintain homogeneity. The obscuration was kept within the optimal range of 8–12% by adjusting the sample concentration. The refractive indices used for Cu@Ag and Al@Ag were 1.52 and 1.36, respectively. Each sample was measured in triplicate. The average values of D10 (10th percentile diameter, defined as fine particle size), D50 (50th percentile diameter, defined as median diameter), D90 (90th percentile diameter, defined as coarse particle size), volume mean diameter and specific surface area (m^2^/kg) are reported.

#### 2.3.5. EMI SE Performance

For EMI SE measurement, a rectangular specimen (23 mm × 10.3 mm; thickness, 2 mm) was placed into a rectangular waveguide holder. The scattering parameters (S_11_, S_12_, S_21_, and S_22_) of the composites were measured by the waveguide method using the ZNB-20 vector network analyser (Rohde & Schwarz GmbH & Co. KG, Munich, Germany). The electromagnetic wave frequency range was 8.2–12.4 GHz. The total shielding effectiveness (SE_T_), reflection loss (SE_R_), absorption loss (SE_A_), and multiple reflection loss (SE_M_) were calculated according to established formulas [31]:

(1) $$\;\begin{array}{c} SE_{A}=10\log\left(\frac{1-{\left|S_{11}\right|}^{2}}{{\left|S_{21}\right|}^{2}}\right) \end{array}$$

(2) $$\;\begin{array}{c} SE_{R}=10\log\left(\frac{1}{1-{\left|S_{11}\right|}^{2}}\right) \end{array}$$

(3) $$\;\begin{array}{c} SE_{T}=SE_{A}+SE_{R}+SE_{M} \end{array}$$

(4) $$\;\begin{array}{c} R={\left|S_{11}\right|}^{2}={\left|S_{22}\right|}^{2} \end{array}$$

(5) $$\;\begin{array}{c} T={\left|S_{21}\right|}^{2}={\left|S_{12}\right|}^{2} \end{array}$$

(6) $$\;\begin{array}{c} 1=T+A+R \end{array}$$

In Equation (3), SE_M_ can be ignored, while SE_T_ is greater than 10 dB.

## 3. Results and Discussion

### 3.1. The Microstructure of Metal Fillers

As shown in Figure 2a,b, the silver-plated copper powder (Cu@Ag) exhibits a flake-like morphology at different magnifications. The surface area of these flake-like structures ranges from 300 μm^2^ to 900 μm^2^. This structure of Cu@Ag enhances the inter-particle contact area, facilitating the formation of an efficient conductive network, thereby promising to improve the electrical conductivity of the composite. The elemental mapping in Figure 2c,d confirms the uniform distribution of copper and silver elements, indicating successful silver plating on the copper flakes. This flake-like structure of Cu@Ag enhances the inter-particle contact area, facilitating the formation of efficient conductive networks and thereby improving the electrical conductivity of the composite material.

In Figure 2e,f, the silver-plated aluminium powder (Al@Ag) exhibits a spherical or ellipsoidal morphology, with a size distribution spanning 10 μm to ~50 μm. The elemental mapping in Figure 2g,h demonstrates uniform silver coating on the aluminium particles. The broad particle size distribution of Al@Ag provides advantages for enhancing the electrical conductivity of the composite. A size-differential filler system wherein smaller particles occupy interstitial spaces between larger particles was used to establish continuous conductive networks.

The distinct morphological differences between Cu@Ag and Al@Ag fillers significantly influence their dispersion behaviour in the composites, as shown in Figure 2i,j. At the same filler loading of 150 phr, the flake-like Cu@Ag particles are difficult to observe in cross-sectional views due to their alignment within the matrix, whereas the spherical/ellipsoidal Al@Ag particles are clearly visible and uniformly distributed throughout the composite, as indicated in Figure 2j. These morphological characteristics are expected to significantly affect the percolation threshold and electromagnetic shielding performance in the silicone rubber matrix. The high aspect ratio of flake-like Cu@Ag particles should facilitate percolation at lower loadings compared to spherical Al@Ag particles, which typically require higher loadings to achieve conductive network connectivity due to their lower aspect ratio and point-contact geometry.

As shown in Figure 2k, the two fillers exhibited distinct particle size characteristics. The Al@Ag filler had a median particle diameter D50 of 47.5 μm, with fine-particle-size D10 and coarse-particle-size D90 values of 29.1 μm and 78.1 μm, respectively. Its volume mean diameter was 51.0 μm, and the specific surface area was 128.4 m^2^/kg. In contrast, the Cu@Ag filler was significantly finer, exhibiting a D50 of 20.1 μm and a narrower distribution (D10 = 7.60 μm, D90 = 43.8 μm). The volume mean diameter for Cu@Ag was 23.4 μm, and it possessed a much larger specific surface area of 393.6 m^2^/kg, which agrees with its flake-like morphology, as observed in Figure 2a.

### 3.2. Effect of Filler Loadings on Performance of Silicone Composites

#### 3.2.1. Physical and Mechanical Properties

**Figure 3:**
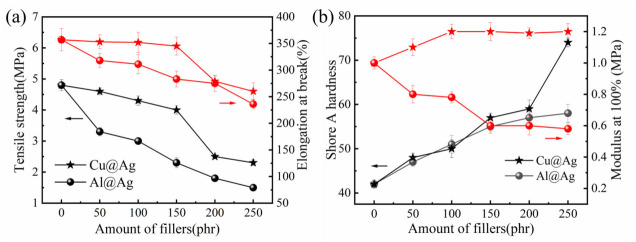
(**a**) Tensile strength (in black, left y-axis) and elongation at break (in red, right y-axis); (**b**) Shore A hardness (in black, left y-axis) and modulus at 100% elongation (in red, right y-axis). Star symbols represent Cu@Ag fillers; solid circles represent Al@Ag fillers.

Figure 3 illustrates the influence of metal filler loadings on the physical and mechanical properties of silicone rubber composites. As shown in Figure 3a, both the tensile strength and elongation at break of the composites gradually decrease with increasing filler loading. This behaviour is attributed to the declining rubber fraction at higher filler loadings, which progressively reduces composite toughness and elasticity. Compared to Al@Ag/silicone composites however, Cu@Ag/silicone composites exhibit superior mechanical performance. In Cu@Ag/silicone composites, the tensile strength and elongation at break remain unchanged or show only a slight decrease at filler loadings up to 150 phr, while these properties decline rapidly above 150 phr. In contrast, the mechanical properties of Al@Ag/silicone composites exhibit a continuous decline within the tested loading range. This difference is attributable to the distinct morphologies of the fillers, as shown in Figure 2. The irregular flake-like structure can form physical interlocking with the silicone rubber matrix, enhancing interfacial adhesion through mechanical anchorage effects. This morphology-dependent interfacial interaction is directly evidenced by the aligned flake microstructure observed in the cross-sectional SEM images (Figure 2i). As a result, Cu@Ag/silicone composites exhibit superior retention of tensile strength and elongation at break at filler loadings up to 150 phr. In contrast, the smooth spherical surface geometry of Al@Ag fillers limits physical interlocking with the matrix, resulting in weaker interfacial adhesion and a continuous decline in mechanical properties from the onset of filler addition. However, when filler content exceeds the critical percolation threshold, negative effects such as filler agglomeration, disruption of matrix continuity, and intensified stress concentration become significantly amplified. These detrimental impacts ultimately outweigh the positive contribution from interfacial enhancement, thus causing a performance decline in composite materials. The spherical/elliptical morphology of the Al@Ag filler inherently limits interfacial adhesion with the rubber matrix, resulting in a performance decrease with increasing filler loading from the onset.

Additionally, at filler loadings up to 200 phr, hardness of the composites shows similar increasing trends with loading, while Cu@Ag/silicone displays rapid hardening above 200 phr, as shown in Figure 3b. The 100% modulus (stress at 100% elongation) of Cu@Ag/silicone composites exhibits an initial rise followed by stabilization, while that of Al@Ag/silicone composites shows a progressive decline. This enhancement is attributed to the flake-shaped configuration of Cu@Ag, which significantly improves composite hardness and elastic modulus through its distinctive two-dimensional geometry and enhanced interfacial interactions.

#### 3.2.2. Thermal Behaviour

**Figure 4:**
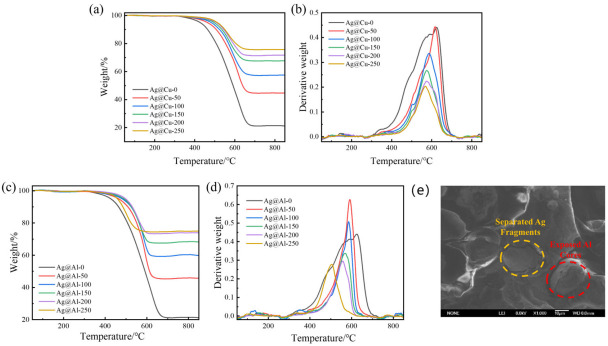
Effect of metal filler loadings on thermal stability of composites. (**a**) TG curve of Cu@Ag/ silicone composites, (**b**) DTG curve of Cu@Ag/ silicone composites, (**c**) TG curve of Al@Ag/ silicone composites, (**d**) DTG curve of Al@Ag/ silicone composites, and (**e**) Ag plating detachment of Al@Ag fillers at 250 phr.

Figure 4 and Table 1 present thermogravimetric analysis (TGA) data composites with varying metal filler loadings. As the Cu@Ag and Al@Ag filler loadings increase, the thermal stability of the composites generally improves. The thermogravimetric data in Figure 4a,c reveal that with increasing filler loading, the initial decomposition temperature (T_d_) and temperatures at 5% and 20% weight loss (T_5_ and T_20_) exhibit upward trends, while the temperature at the maximum decomposition rate (T_max_) decreases. The T_d_, T_5_, and T_20_ of the Cu@Ag composite with a filler loading of 250 phr increase by 90 °C, 85.8 °C, and 98.9 °C, respectively, compared with the control sample (Cu@Ag-0), while the T_max_ decreases by 55.1 °C. The thermal stability enhancement observed with increasing metallic filler loading is attributed to multiple factors. The well-dispersed metallic filler network restricts the segmental mobility of silicone rubber molecular chains, impeding the initiation of thermal degradation and raising the onset decomposition temperature. Additionally, the dense filler network acts as a physical barrier that hinders the diffusion and release of volatile thermal decomposition products from the composite matrix, thereby retarding the overall decomposition process [32]. The intrinsically high thermal stability of the metallic fillers further contributes by diluting the thermally labile silicone rubber phase at higher loadings. For the Al@Ag/silicone composite, T_d_, T_5_, and T_20_ values show similar stable increases (Δ + 78 °C, +71.2 °C, +60.2 °C) at loadings ≤ 200 phr, while dropping dramatically to near-unfilled control sample levels at 250 phr loading, as shown in Table 1. Exceeding the critical loading threshold (250 phr for Al@Ag) causes rapid performance deterioration likely due to poor filler dispersion and matrix disruption at excessive loadings, as shown in Figure 4e. The images clearly show that the Ag plating on Al microspheres (indicated by the red dashed circle) has peeled off, resulting in two distinct phenomena: (a) exposed Al cores and (b) separated Ag fragments (indicated in the orange dashed circle) dispersed within the rubber matrix. This indicates that the mechanical integrity of the core–shell structure was compromised during processing at this high loading [33]. At 250 phr, the excessive filler content drastically increases filler–filler contact and shear forces during mixing. These elevated shear forces are believed to mechanically damage the Ag coating on the Al microspheres, promoting coating detachment, as evidenced by the SEM images in Figure 4e. The detached Ag fragments and exposed Al cores then form agglomerates and defects, which may act as sites for accelerated thermal-oxidative degradation.

#### 3.2.3. Electrical Conductivity

**Figure 5:**
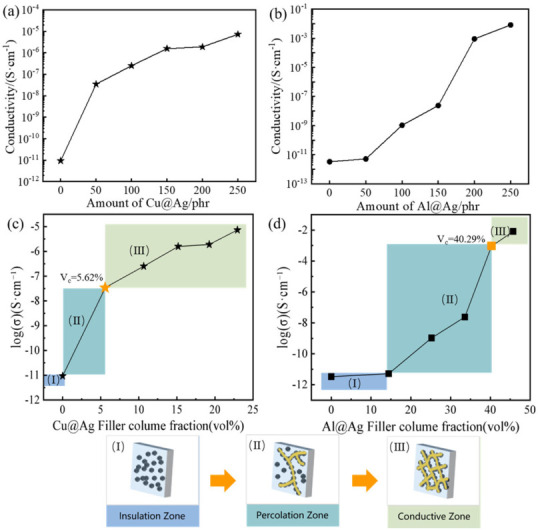
Electrical conductivity as a function of filler loading for (**a**) Cu@Ag/silicone composites and (**b**) Al@Ag/silicone composites. The y-axis in (**a**) spans from 10^−12^ to 10^−4^ S·cm^−1^ and in (**b**) spans from 10^−13^ to 10^−1^ S·cm^−1^. At 200 phr, the conductivity of Cu@Ag/silicone is approximately 10^−6^ S·cm^−1^ and that of Al@Ag/silicone is approximately 10^−3^ S·cm^−1^. Logarithm of electrical conductivity (log σ) as a function of filler volume fraction for the Cu@Ag/silicone (**c**) and Al@Ag/silicone (**d**) composites. Stages (**I**)–(**III**) denote Insulation, Percolation and Conductive Zone, respectively.

Figure 5 illustrates the effect of filler loading on the electrical conductivity of both composites. The electrical conductivity of Cu@Ag/silicone composites increases dramatically at the beginning when the filler loading is low, as illustrated in Figure 5a. At 50 phr Cu@Ag, the conductivity rises by approximately four orders of magnitude compared to the unfilled control sample (from ~10^−12^ to ~10^−8^ S·cm^−1^), indicating the onset of electrical percolation. It is the beginning of incipient conductive network formation rather than a fully established continuous network which conventionally requires conductivity ≥ 10^−6^ S·cm^−1^. Full electrical percolation for Cu@Ag is therefore more accurately placed between 50 and 100 phr, where the conductivity crosses this threshold, attributed to the high-aspect-ratio flake-like morphology that provides large contact areas and promotes efficient conductive network formation. When the loading increases to 250 phr, the conductivity increases by only two additional orders of magnitude compared to 50 phr, consistent with a saturating network density above the percolation region.

For Al@Ag/silicone composites (Figure 5b), however, electrical conductivity increases more gradually with loading. At 50 phr Al@Ag, the conductivity shows negligible improvement. At 100 phr, the conductivity increases by two orders of magnitude, and at 200 phr, it rises by nine orders of magnitude compared to the control sample at 0 phr. As supported by Figure 2, the higher percolation threshold (approximately 200 phr) for Al@Ag is attributed to the spherical morphology, which primarily exhibits point contacts within the rubber matrix, resulting in lower contact efficiency and requiring higher loadings to establish effective conductive networks.

Figure 5c presents the logarithm of electrical conductivity (log σ) as a function of filler volume fraction for Cu@Ag/silicone composites, and Figure 5d shows the corresponding data for Al@Ag/silicone composites. The filler volume fraction (V) was converted from the phr loading using the relation V = (W_f_/ρ_f_)/(W_f_/ρ_f_ + W_r_/ρ_r_) × 100%, where W_f_ and W_r_ are the weights of the filler and silicone rubber matrix (100 g), and ρ_f_ and ρ_r_ are the densities of the filler and silicone rubber (1.1 g/cm^3^), respectively. Based on the rule of mixtures, the density of the Cu@Ag filler (20 wt% Ag) was estimated to be approximately 9.23 g/cm^3^, while that of the Al@Ag filler (23 wt% Ag) was approximately 3.26 g/cm^3^.

The curves for both composites clearly exhibit three distinct stages of conductive network evolution. In Stage I, the insulating region, the filler content lies below the percolation threshold (V_c_); the conductive particles are isolated; and the conductivity remains negligible. Stage II, the percolation region, commences when V exceeds V_c_. For the Cu@Ag composite, the percolation threshold is 5.62 vol% (equivalent to 50 phr), whereas for the Al@Ag composite it is approximately 40.29 vol% (equivalent to 200 phr). At these critical loadings, the first continuous conductive pathway forms, causing a sharp increase in conductivity. As V increases further, the system enters Stage III, the saturation region, where the network becomes dense and a multi-pathway, and the conductivity growth gradually levels off. The markedly lower percolation threshold of Cu@Ag relative to Al@Ag is primarily attributed to the high-aspect-ratio flake-like morphology of the Cu@Ag particles, which enables long-range electrical contacts at very low filler loadings, while the near-spherical Al@Ag particles require much denser packing to achieve percolation.

#### 3.2.4. EMI Shielding Effectiveness

**Figure 6:**
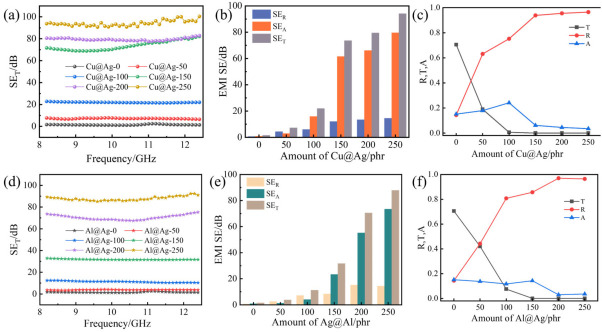
The effect of metal filler loading on the electromagnetic shielding performance of the composites: (**a**) EMI SE of Cu@Ag/silicone composites; (**b**) SE_R_, SE_A_, and SE_T_ of Cu@Ag/silicone composites; (**c**) EMI shielding coefficient of Cu@Ag/silicone composites at 8.2 GHz; (**d**) EMI SE of Al@Ag/silicone composites; (**e**) SE_R_, SE_A_, and SE_T_ of Al@Ag/silicone composites; (**f**) EMI shielding coefficient of Al@Ag/silicone composites at 8.2 GHz.

Figure 6 demonstrates the loading-dependent EMI SE properties of Cu@Ag/silicone and Al@Ag/silicone composites. As shown in Figure 6a, the SE of Cu@Ag/silicone composites increases progressively with filler loading, rising from ~1 dB at 0 phr to ~70 dB at 150 phr. The critical loading for practical EMI shielding performance (defined here as the loading above which a step-change in EMI SE is observed and performance exceeds practically relevant levels) is approximately 150 phr for Cu@Ag, which is distinct from and higher than the electrical percolation onset (~50 phr) and full electrical percolation (~50–100 phr). This is consistent with the literature, where the critical loading for effective EMI shielding typically exceeds the electrical percolation threshold because shielding effectiveness depends not only on network connectivity but also on the overall conductivity magnitude and available reflection/absorption cross-section of the filler network. For Al@Ag, the critical loading for practical EMI shielding performance is approximately 200 phr, as shown in Figure 6d, consistent with its higher electrical percolation threshold arising from the spherical morphology and lower aspect ratio.

As shown in Figure 6b,e, the reflection loss (SE_R_) of both Cu@Ag/silicone and Al@Ag/silicone composites remains relatively constant with increasing filler content, while the absorption loss (SE_A_) gradually increases. At equivalent filler loadings, Cu@Ag/silicone composites exhibit higher SE_R_ and SE_A_ values compared to Al@Ag/silicone composites. Consequently, Cu@Ag/silicone composites demonstrate superior total shielding effectiveness (SE_T_), particularly at intermediate filler loadings of 100 and 150 phr. The superior low-loading performance of Cu@Ag/silicone composites is attributed to the lamellar structure of Cu@Ag, which exhibits a high aspect ratio and large surface area, facilitating the formation of overlapping conductive pathways within the rubber matrix. Additionally, the multiple interfaces between Cu@Ag layers enhance interfacial polarization, contributing to a high SE at relatively low filler loadings.

Figure 6c,f present the electromagnetic shielding power coefficients for both composite systems with varying metal filler loadings. The absorption coefficient (A), reflection coefficient (R), and transmission coefficient (T) are used to quantitatively assess the electromagnetic wave absorption and reflection capabilities of these shielding materials. At 0 phr filler loading, the transmission coefficient (T) is high (approximately 0.7), indicating negligible electromagnetic shielding capability with 70% of incident electromagnetic waves passing through the composite. As the metal filler loading increases, the reflection coefficient (R) of both composites rises significantly. At 250 phr, the reflection coefficient reaches approximately 0.96, suggesting that both Cu@Ag/silicone and Al@Ag/silicone composites primarily function as reflection-dominated electromagnetic shielding materials. This enhancement is attributed to the formation of robust conductive pathways within the rubber matrix at higher filler loadings, which significantly improves the composite’s electrical conductivity, increases the impedance mismatch with air, and consequently elevates the reflection coefficient.

### 3.3. Properties of Sandwich-Structured Composite Materials

Based on the single-layer composite performance, Cu@Ag/silicone and Al@Ag/silicone composites, each containing 200 phr of respective fillers, were selected as the constituent layers for sandwich-structured materials. The sandwich structures were fabricated through pre-forming and co-vulcanization processes, with the total thickness controlled at approximately 2 mm. Four material configurations were prepared and designated as follows: M-1 (single-layer Cu@Ag/silicone), M-2 (single-layer Al@Ag/silicone), M-3 (Al@Ag-Cu@Ag-Al@Ag sandwich), and M-4 (Cu@Ag-Al@Ag-Cu@Ag sandwich).

#### 3.3.1. Morphological Structure

**Figure 7:**
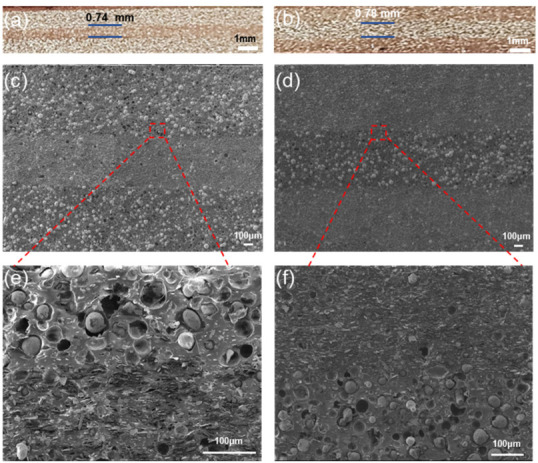
Morphological characterisation of sandwich-structured composites. (**a**,**b**) Optical microscope images of M-3 (Al@Ag-Cu@Ag-Al@Ag) and M-4 (Cu@Ag-Al@Ag-Cu@Ag) cross-sections with intermediate layer thickness measurement; (**c**,**d**) cross-sectional SEM images of M-3 and M-4 revealing the sandwich structure; (**e**,**f**) high-magnification SEM images of the interfacial regions highlighted in panels (**c**,**d**), respectively.

Figure 7 presents the cross-sectional morphology of the sandwich-structured composites. The optical microscope images in Figure 7a,b clearly reveal the distinct layered architecture of both M-3 and M-4 configurations. The intermediate layers have thicknesses of 0.74 mm and 0.76 mm for M-3 and M-4, respectively, demonstrating good control over layer thickness during the fabrication process.

High-magnification SEM images (Figure 7c,d) demonstrate that the layers are continuous and uniform with well-defined boundaries. The intermediate layer thickness distribution is even, indicating successful pre-forming and co-vulcanization processes. Figure 7e,f show the interfacial bonding between Cu@Ag/silicone and Al@Ag/silicone layers, revealing excellent interfacial adhesion with no apparent defects or delamination. The tight bonding between the two-phase interface is crucial for mechanical integrity and effective load transfer in the sandwich structures.

#### 3.3.2. Mechanical Properties

**Figure 8:**
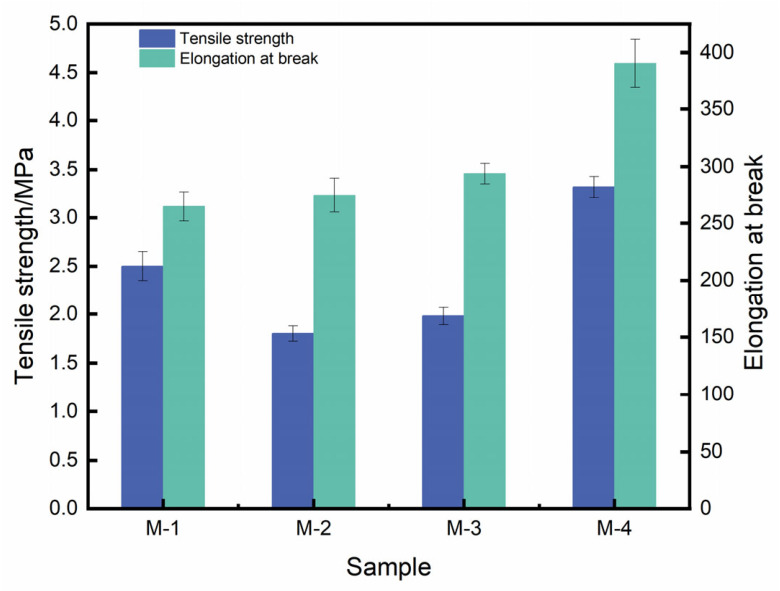
Tensile and elongation at break of single-layer (M-1 and M-2) and sandwich-structured (M-3 and M-4) composites. Tensile strength (dark blue bars, left y-axis) and elongation at break (dark green bars, right y-axis).

Figure 8 presents the physical and mechanical properties of single-layer and sandwich-structured composites. The tensile strength of the single-layer Cu@Ag/silicone composite (M-1) is slightly higher than that of the Al@Ag/ silicone composite (M-2), primarily attributed to the spherical morphology of Al@Ag particles, which are more prone to forming defects at the same filler loading (200 phr). Compared with M1 and M2, the sandwich-structured composites M-3 (Al@Ag-Cu@Ag-Al@Ag) and M-4 (Cu@Ag-Al@Ag-Cu@Ag) exhibit significantly higher elongation at break.

The tensile strength of M-3 falls between those of M-1 and M-2, representing an intermediate behaviour consistent with its composite structure. Notably, M-4 exhibits an improvement in tensile strength over M-1. This is attributed to the outer Cu@Ag/silicone layers in the M-4 configuration, which bear the primary tensile stress during deformation. Their dominant load-bearing role contributes to the improved overall tensile strength of the sandwich composite. The enhanced mechanical properties in sandwich structures indicate that the combination of different filler morphologies creates a more balanced stress distribution compared to single-filler systems. As studied in Figure 8, interfacial bonding between layers provides additional resistance to crack propagation. The successful interfacial bonding is attributed to the co-vulcanization process, which ensures chemical cross-linking between layers during curing, creating strong covalent bonds at the interfaces.

The enhanced mechanical properties of sandwich structures arise from several synergistic mechanisms. The significantly improved elongation at break in both M-3 and M-4 compared to single-layer composites results from the multilayer architecture creating heterogeneous stress distribution during deformation. The Cu@Ag-rich and Al@Ag-rich layers possess different elastic moduli. This modulus mismatch prevents premature stress concentration within any single layer. Furthermore, the interfaces between morphologically distinct layers act as crack deflection sites. Propagating cracks are forced along tortuous paths at these interfaces, increasing energy dissipation before failure [34]. Additionally, spherical Al@Ag particles can undergo localized rotation under stress, providing deformation mechanisms unavailable in purely flake-filled systems [35]. The superior tensile strength of M-4 (Cu@Ag-Al@Ag-Cu@Ag) compared to M-1 reflects the strategic advantage of the outer-layer configuration. The outer Cu@Ag/silicone layers possess stronger matrix–filler interfacial adhesion through mechanical interlocking from flake morphology [36,37]. These layers directly bear primary tensile stress and protect the inner Al@Ag layer with inherently weaker spherical filler–matrix interfaces. This configuration principle resembles fibre-reinforced laminates where stronger layers occupy stress-bearing positions. Conversely, M-3 with weaker Al@Ag outer layers exhibits intermediate strength. The co-vulcanization process ensures strong interfacial bonding through chemical cross-linking, enabling effective stress transfer and preventing delamination, as confirmed by SEM analysis (Figure 7e,f).

#### 3.3.3. Electrical Conductivity

**Figure 9:**
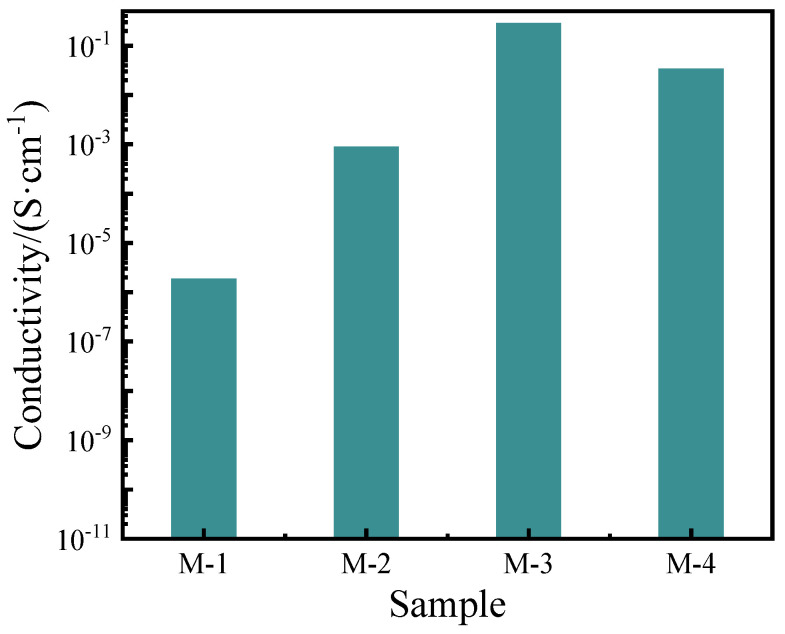
Effect of interlayer ordering on the conductivity property of composites M1-M4 at 200 phr filler loading.

Figure 9 illustrates the effect of sandwich structure on the electrical conductivity of the composites. Interestingly, the electrical conductivity of the single-layer Cu@Ag/silicone composite (M-1) at 200 phr is 10^−6^ S·cm^−1^, significantly lower than that of the Al@Ag/silicone composite (M-2) at 10^−3^ S·cm^−1^. This apparent contradiction to the earlier percolation behaviour (where Cu@Ag showed a lower percolation threshold) is attributed to the specific filler loading (200 phr) and the distinct conduction mechanisms of the two morphologies. At 200 phr loading, the lamellar morphology of Cu@Ag results in preferential parallel stacking within the rubber matrix, which can reduce the effective contact area for electron transport in the through-thickness direction. In contrast, the spherical morphology of Al@Ag at this high loading (which exceeds its percolation threshold) facilitates three-dimensional conductive pathways through multiple point contacts, yielding higher bulk electrical conductivity.

Both sandwich-structured composites M-3 (Al@Ag-Cu@Ag-Al@Ag) and M-4 (Cu@Ag-Al@Ag-Cu@Ag) exhibit electrical conductivities higher than their single-layer counterparts. This enhancement is attributed to the formation of more complex and robust conductive networks within the multilayer structure. The interfaces between different layers create additional pathways for electron transport, and the combination of flake-like and spherical fillers provides complementary conduction mechanisms that enhance overall conductivity.

#### 3.3.4. EMI Shielding Effectiveness

**Figure 10:**
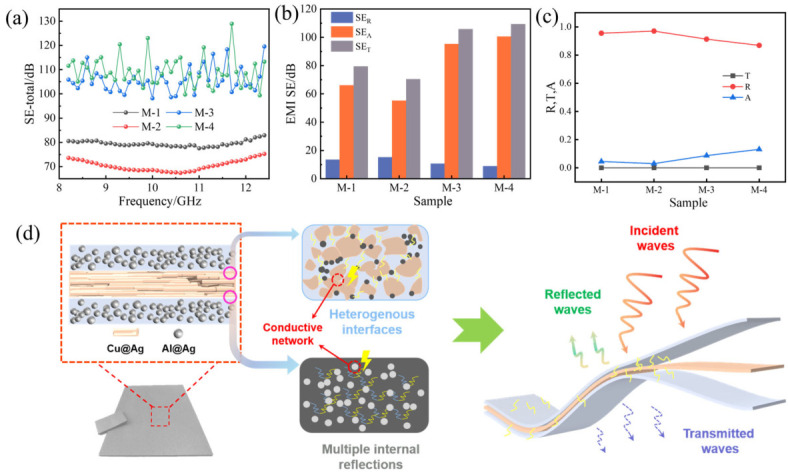
The effect of sandwich structure on the electromagnetic shielding performance of composites. (**a**) EMI SE of laminated composites. (**b**) SE_R_, SE_A_, and SE_T_ of laminated composites. (**c**) EMI shielding coefficient of laminated composites at 8.2 GHz. (**d**) Proposed conceptual schematic of the EMI shielding mechanism in sandwich-structured composites, illustrating multiple reflections and absorption losses at morphologically distinct layer interfaces. This diagram represents a qualitative mechanistic framework consistent with the S-parameter data presented in panels (**b**,**c**); quantitative separation of interfacial contributions requires future finite element modelling or layer-resolved impedance measurements.

Figure 10 demonstrates the superior electromagnetic shielding performance of sandwich-structured composites. As shown in Figure 10a, the single-layer Cu@Ag/silicone composite (M-1) at 200 phr exhibits an EMI SE of approximately 80 dB, while the Al@Ag/silicone composite (M-2) achieves approximately 70 dB at the same filler loading. Remarkably, both sandwich-structured composites M-3 (Al@Ag-Cu@Ag-Al@Ag) and M-4 (Cu@Ag-Al@Ag-Cu@Ag) demonstrate significantly enhanced EMI SEs, averaging around 110 dB. As observed in Figure 10b, all four laminated composites exhibit similar reflection loss, SE_R_. However, the absorption loss, SE_A_, of sandwich-structured composites M-3 and M-4 is significantly greater than that of single-layer composites M-1 and M-2. This indicates that the layered architecture substantially enhances the electromagnetic wave absorption capability of the composite.

Compared with reported single and multilayer EMI shielding materials, as listed in Table 2, our sandwich structures demonstrate comparable performance with previously reported silicone-based composites. The 33% improvement over single-layer composites and the achievement of an ~110 dB EMI SE represents a significant advancement, demonstrating the effectiveness of the multilayer design approach. The specific shielding effectiveness (SSE/t) values were calculated using the formula SSE/t = EMI SE/(thickness × density), which provides a fair comparison independent of sample dimensions and material density. As shown in Table 2, our Cu@Ag-Al@Ag sandwich structures achieve an approximately 110 dB EMI SE at an only 2 mm thickness, with a calculated SSE/t of 297 dB·cm^2^·g^−1^. This performance substantially exceeds that of previously reported silicone-based multilayer systems, such as graphene/silicone sandwiches and Fe_3_O_4_@CNT/NR sandwiches. Although the Ag@GMs/silicone composite (120 dB) shows a higher absolute EMI SE than our sandwich composite (110 dB), its SSE/t values are comparable (~327.9 vs. ~297.3 dB·cm^2^·g^−1^). While ultra-lightweight materials such as aerogels and foams can achieve significantly higher SSE/t values (e.g., >10,000 dB·cm^2^·g^−1^) due to their extremely low densities, such materials often lack the mechanical robustness and processability required for practical applications. Within the category of metal-plated filler/polymer composites, our materials demonstrate competitive or superior normalized performance while offering additional advantages of mechanical flexibility, excellent thermal stability (Td > 450 °C), and processability via conventional rubber processing techniques.

Analysis of the shielding mechanisms reveals that all four samples, M1-M4, exhibit significantly higher reflection coefficients than absorption coefficients, indicating reflection-dominated shielding behaviour at layer interfaces. Importantly, the sandwich structures M-3 and M-4 show notably higher absorption coefficients compared to their single-layer counterparts M-1 and M-2 (Figure 10c), which is directly evidenced by the S-parameter measurements. This experimentally observed enhancement in SE_A_ is consistent with a multiple-reflection and conductivity-gradient mechanism, as illustrated conceptually in Figure 10d. Impedance mismatches at morphologically distinct layer interfaces are expected to cause multiple internal reflections that increase electromagnetic wave path length and energy dissipation, while the conductivity gradient across layers may induce additional eddy current losses and polarisation losses at heterogeneous filler–polymer interfaces. However, we acknowledge that direct quantitative separation of the contribution of each individual interface, for example through finite element modelling of the field distribution or layer-resolved impedance measurements, was beyond the scope of this study. The mechanistic diagram in Figure 10d should therefore be understood as a conceptual framework consistent with the observed data, rather than a quantitatively verified account. Future work employing finite element electromagnetic simulations or transfer matrix modelling of the layered impedance profile would enable rigorous quantitative validation of the proposed mechanism.

The superior performance of sandwich structures demonstrates that architectural design can be as important as material composition in developing high-performance EMI shielding materials. The developed sandwich-structured composites show significant potential for practical EMI shielding applications in flexible electronics. The combination of a high EMI SE (~110 dB), excellent thermal stability (Td > 450 °C), and mechanical flexibility make these materials particularly suitable for: (1) aerospace and automotive electronics, such as in high-temperature environments requiring both EMI protection and thermal stability [38]; (2) wearable and portable electronic devices, such as in lightweight, flexible EMI shielding for 5G communication devices, smartwatches, and medical sensors [39,40,41]; (3) flexible circuit boards and connectors, such as in conformable shielding gaskets for electronic housings and cable assemblies [42]; and (4) IoT devices, such as in compact electronic systems requiring efficient broadband EMI protection in the X-band and beyond [4]. In summary, the combination of different filler morphologies and a multilayer design creates synergistic effects that exceed the performance of individual components, offering additional advantages of tuneable impedance matching and frequency-selective shielding for various potential applications.

Regarding practical application perspectives, the X-band frequency range (8.2–12.4 GHz) evaluated in this study is widely adopted as the standard benchmarking range in the EMI shielding literature and encompasses frequencies relevant to radar systems, satellite communications, and certain 5G mid-band applications. However, it is acknowledged that direct extrapolation of the X-band shielding performance to other frequency regimes, including sub-GHz bands (0.1–1 GHz) relevant to IoT, RFID, and 4G LTE devices; the 2.4 GHz and 5 GHz Wi-Fi and Bluetooth bands; and the millimetre-wave 5G bands (24–40 GHz), is not straightforward due to the frequency dependence of skin depth, permittivity, and conductivity contributions to shielding effectiveness. A broadband characterisation across sub-GHz to millimetre-wave frequencies is proposed as a priority direction for future work to fully establish the practical applicability of these sandwich-structured composites across the frequency ranges relevant to modern telecommunication and consumer electronic applications.

## 4. Conclusions

This study provides a systematic comparison of flake-like Cu@Ag and spherical Al@Ag silver-plated fillers in silicone rubber matrices and demonstrates a novel sandwich architecture that synergistically combines morphologically distinct fillers to achieve exceptional EMI shielding performance. The key conclusions are as follows:

(1) Filler morphology governs percolation behaviour: The flake-like Cu@Ag filler exhibits electrical percolation onset at ~50 phr, with full electrical percolation established between 50 and 100 phr, owing to its high aspect ratio and large inter-particle contact area. The spherical Al@Ag filler reaches full electrical percolation at approximately 200 phr due to its lower aspect ratio and point-contact geometry.
(2) Distinct critical loadings for practical EMI shielding performance: The critical loading for practical EMI shielding performance is approximately 150 phr for Cu@Ag and 200 phr for Al@Ag composites, which is distinct from and higher than the respective electrical percolation thresholds. This distinction reflects the additional conductivity magnitude required for effective electromagnetic wave attenuation, and the two thresholds should not be conflated.
(3) Both filler systems achieve a high EMI SE at maximum loading: At 250 phr filler loading, both Cu@Ag/silicone and Al@Ag/silicone composites achieve comparable EMI shielding effectiveness exceeding 90 dB across the X-band frequency range (8.2–12.4 GHz), meeting the requirements for most practical shielding applications.
(4) Sandwich structures deliver significant improvement over single-layer composites: The multilayer architectures (Al@Ag-Cu@Ag-Al@Ag designated M-3, and Cu@Ag-Al@Ag-Cu@Ag designated M-4) achieved an EMI shielding effectiveness of approximately 110 dB at 2 mm total thickness, representing a 33% improvement over the best single-layer composite. The specific shielding effectiveness (SSE/t) of ~297 dB·cm^2^·g^−1^ substantially exceeds previously reported silicone-based multilayer systems.
(5) Enhancement mechanism of sandwich structures: The improved performance of sandwich structures is attributed to multiple electromagnetic wave interactions at morphologically distinct layer interfaces and enhanced absorption through conductivity gradients. This mechanistic framework is consistent with the S-parameter data but requires future validation through finite element electromagnetic simulations or transfer matrix modelling.
(6) Thermal stability and mechanical flexibility: Both single-layer and sandwich-structured composites demonstrate excellent thermal stability (Td > 450 °C) and retain mechanical flexibility, making them suitable for demanding applications in aerospace, automotive electronics, and wearable devices.
(7) Future work directions: (i) conduct a broadband EMI shielding characterisation beyond the X-band to establish applicability across telecommunication and consumer device frequency ranges; (ii) investigate long-term stability under cyclic deformation, accelerated thermal ageing, humidity, and corrosive environments; and (iii) employ finite element modelling or layer-resolved impedance measurements to quantitatively validate the proposed sandwich-structure shielding mechanism.

## Figures and Tables

**Table 1:** TGA data of the effect of metal filler amount on thermal stability of composites.

Sample	T_d_/°C	T_5_/°C	T_20_/°C	T_max_/°C	CR700/%
Cu@Ag-0	373.0	426.8	507.1	623.9	21.2
Cu@Ag-50	399.6	463.0	553.6	616.9	44.6
Cu@Ag-100	416.1	482.5	568.8	587.0	57.3
Cu@Ag-150	435.3	496.5	579.1	576.5	67.5
Cu@Ag-200	446.1	508.0	592.6	576.9	71.3
Cu@Ag-250	463.0	512.6	606.0	568.8	75.6
Al@Ag-50	403.3	464.6	551.0	591.9	45.3
Al@Ag-100	404.5	481.3	562.5	586.3	59.3
Al@Ag-150	433.0	489.7	562.8	568.8	67.6
Al@Ag-200	451.0	498.0	567.3	557.5	73.4
Al@Ag-250	413.6	439.5	502.3	469.5	77.5

**Table 2:** Performance comparison with reported EMI shielding materials.

Material System	Structure	Thickness (mm)	Density (g/cm^3^)	EMI SE (dB)	Frequency (GHz)	SSE/t (dB·cm^2^·g^−1^)	Ref.
**Single-layer metal-filled composites**							
Ag@GMs/silicone	Single layer	2	~1.83	~120	8–12	~327	[20]
Ni-CF/silicone	Single layer	-	~2.0	>80	0.03–1.2	~40	[21]
**Cu@Ag/silicone (this work)**	**Single layer**	**2**	**1.95**	**~80**	**8.2–12.4**	**~205**	**-**
**Al@Ag/silicone (this work)**	**Single layer**	**2**	**1.75**	**~70**	**8.2–12.4**	**~200**	**-**
**Multilayer-structured composites**							
Fe_3_O_4_@CNT/NR sandwich	3 layers	~3	~1.2	~45	8.2–12.4	~125	[25]
Graphene/silicone sandwich	3 layers	2	~1.1	30.4	1–3	~138	[26]
MXene/Fe_3_O_4_ multilayer	Multilayer	-	-	~50	8.2–12.4	-	[22]
**Cu@Ag-Al@Ag sandwich (this work)**	**3 layers**	**2**	**1.85**	**~110**	**8.2–12.4**	**~297**	**-**

## Data Availability

The data presented in this study are openly available in FigShare at https://doi.org/10.6084/m9.figshare.32145451, accessed on 12 July 2026.
